# Increased microbial loading in aerosols produced by non-contact air-puff tonometer and relative suggestions for the prevention of coronavirus disease 2019 (COVID-19)

**DOI:** 10.1371/journal.pone.0240421

**Published:** 2020-10-08

**Authors:** Hui Guo, Wei Li, Yingying Huang, Xiaoyan Li, Zhi Li, Hongxia Zhou, Enhua Sun, Li Li, Jisheng Li

**Affiliations:** 1 Department of Ophthalmology, Qilu Hospital, Cheeloo College of Medicine, Shandong University, Jinan, Shandong Province, China; 2 Department of Clinical Laboratory, Qilu Hospital, Cheeloo College of Medicine, Shandong University, Jinan, Shandong Province, China; 3 Department of Medical Oncology, Qilu Hospital, Cheeloo College of Medicine, Shandong University, Jinan, Shandong Province, China; University of the Pacific - Arthur A Dugoni School of Dentistry, UNITED STATES

## Abstract

**Objective:**

To evaluate the microbial loading in aerosols produced after air-puff by non-contact tonometer (NCT) as well as the effect of alcohol disinfection on the inhibition of microbes and thus to provide suggestions for the prevention and control of COVID-19 in ophthalmic departments of hospitals or clinics during the great pandemics.

**Methods:**

A cross-sectional study was carried out in this study. A NIDEK NCT was used for intraocular pressure (IOP) measurement for patients who visited Department of Ophthalmology in Qilu Hospital of Shandong University during March 18–25 2020. After ultra-violate (UV) light disinfection, the room air was sampled for 5 minutes. Before and after alcohol disinfection, the air samples and nozzle surface samples were respectively collected by plate exposure method and sterile moist cotton swab technique after predetermined times of NCT air-puff. Microbial colony counts were calculated after incubation for 48 hours. Finally, mass spectrometry was performed for the accurate identification of microbial species.

**Results:**

Increased microbial colonies were detected from air samples close to NCT nozzle after air-puff compared with air samples at a distance of 1 meter from the nozzle (*p* = 0.001). Interestingly, none microbes were detected on the surface of NCT nozzle. Importantly, after 75% alcohol disinfection less microbes were detected in the air beside the nozzle (*p* = 0.003). Microbial species identification showed more than ten strains of microbes, all of which were non-pathogenic.

**Conclusion:**

Aerosols containing microbes were produced by NCT air-puff in the ophthalmic consultation room, which may be a possible virus transmission route in the department of ophthalmology during the COVID-19 pandemic. Alcohol disinfection for the nozzle and the surrounding air was efficient at decreasing the microbes contained in the aerosols and theoretically this prevention measure could also inhibit the virus. This will give guidance for the prevention of virus transmission and protection of hospital staff and patients.

## Introduction

At the time of this publication, the coronavirus disease of 2019 (COVID-19), caused by the severe acute respiratory syndrome coronavirus 2 (SARS-CoV-2), is a global public health emergency [1, 2]. SARS-CoV-2 is spread through respiratory droplets and direct contact. Droplet transmission of virus is via larger respiratory particles, generally above 5μm in diameter, which tends to travel no more than one meter. Contact transmission occurs when the virus remains on the surface and therefore becomes a potential source of infection. The SARS-CoV-2 virus has been found to survive on plastic, stainless steel and other surfaces between several hours to several days [3, 4]. Recently, attention has been directed toward the possibility of transmission of SARS-CoV-2 virus through aerosols [5]. In the latest diagnosis and treatment plan issued by the National Health Commission (NHC) of China, "aerosol transmission" was added to the "droplet respiratory particles and close contacts" as one of the major transmission routes of SARS-CoV-2 [6].

Aerosols are composed of solid or liquid particles dispersed and suspended in the air. They contain soil particles, industrial dust particles, bacteria, microorganisms or other components. Aerosols containing pathogens range from 1.0 to 5.0μm in size and can travel hundreds of meters or more. Currently, several studies have shown that aerosols are involved in the spread of severe acute respiratory syndrome (SARS), Middle East respiratory syndrome (MERS), H1N1 flu and other respiratory diseases [7–9]. Van Doremalen et al. have found that SARS-CoV-2 could be transmitted through aerosol and fomite because the virus is viable and infectious in aerosols for hours and on surfaces for several days [3]. Similarly, Wang et al. have reported that COVID-19 might be transmitted through aerosols, based on the epidemiological evidence in some confirmed cases in Wuhan, China [10]. Therefore, the involvement of aerosols in the transmission of the SARS-CoV-2, especially in healthcare institutions requires more attention.

Aerosol generation occurs when air accelerates across a fluid surface. In ophthalmology, non-contact tonometer (NCT), which is commonly used for ophthalmological examination, can generate localized aerosols. Britt et al., using a camera and flash electrically coupled to NCT to photograph the corneal profile, have revealed tear film dehiscence and microaerosol formation in most eyes [11]. Tears have been implicated as a source of virus infection. Some studies have detected virus particles in conjunctival secretions among SARS patients in 2003 [12] and the presence of SARS-CoV-2 in tears and conjunctival secretions in COVID-19 patients with conjunctivitis [13]. It has been reported that SARS-CoV-2 could be transmitted through the mucous membranes, including the conjunctiva [14, 15]. Many ophthalmologists have been infected through routine diagnosis and treatment [14]. Therefore, the aerosols produced by NCT may be one of transmission routes of SARS-CoV-2. A synoptic study by Yu et al. on the guidelines and clinical practice experiences in the departments of ophthalmology in large-scale hospitals in China has stressed the risk of air-puff tonometer in the transmission of the virus [16]. If an asymptomatic COVID-19 patient with an eye condition undergoes an NCT examination, the aerosols containing SARS-CoV-2 may be formed, and thus may be transmitted to healthcare specialists and patients with compromised or no protective gear. However, empirical studies validating this proposition are scarce.

A recent study by Li et al. has used an air quality detector to confirm that NCT could produce aerosols [17]. However, it remains unknown if the aerosols contained microbes after non-contact “air-puff” tonometry. Because the COVID-19 has been successfully controlled in Jinan, China, we could not acquire the air samples containing SARS-CoV-2. The present study was designed to evaluate the microbes in the aerosols produced by non-contact “air-puff” tonometer and to determine the effect of alcohol disinfection on the inhibition of the microbes. This will provide a microbial reference for ophthalmologists, which may be helpful in the control and prevention of COVID-19.

## Materials and methods

### Non-contact air-puffed tonometry

Non-contact air-puffed tonometry is a widely used technology for the clinical measurement of the intraocular pressure (IOP) by a NIDEK NCT. This technology is the quickest and easiest in checking whether a patient has normal or high IOP. It involves the use of air-puff to achieve the desired results since there is no contact between the patient's eye and the NCT. In this study, patients were excluded infectious eye diseases before NCT examination and wore facial masks during the whole course according to the prevention and control management measures of the Qilu Hospital of Shandong University. The IOP of each patient’s eye was measured 3 times, which means 6 times of air-puff for one patient, both eyes. When 1, 5, 10 and 15 patients were given IOP measurement, 6-, 30-, 60- and 90-times of air-puff were finished respectively. All sampling for the four groups classified by times of NCT air-puff was done between March 18–25, 2020. All patients were briefed on the aim of the study and their consent obtained before sampling. The ethics committee of the Qilu Hospital of Shandong University approved this study.

### Sample collection

After disinfecting the room with ultra-violate (UV) light for 15 minutes, the room air was sampled for 5 minutes using plates with blood agar culture medium. The plates were placed in 3 different sites (inner corner, middle and outer corner), at 1 meter high from the floor and in a diagonal line in the ophthalmic consultation room with an area of 15 square meters according to the Hospital Disinfection and Sanitation standard (GB15982-2012) of China [18]. After different times of NCT air-puff, air sampling was done close to the NCT nozzle (10 centimeters in diameter) and at a 1 meter (m) distance from the nozzle at the same height from the floor for 5 minutes. Surface samples from the nozzle were collected using a moist cotton swab technique. Disinfection with 75% alcohol was applied by spraying 75% alcohol on the NCT nozzle and the surrounding air and wiping the nozzle surface using the alcohol after the NCT examination. During the sampling, the door of the consultation room remained open. The sampling was done triplicately on three random days and a total of 45 patients were enrolled in this study.

### Culture and identification

Plates were incubated at 37°C in an anaerobic chamber for 48 hours after sample collection and the total colony counts were obtained. The species of each colony were identified using the matrix-assisted laser desorption/ionization time-of-flight mass spectrometry (MALDI-TOF MS).

### Statistical analysis

A descriptive analysis was performed to evaluate the features of the samples. Microbial density was measured as colony-forming units per plate (cfu/plate). A paired *t*-test was used to compare the microbial density and *p*<0.05 indicated statistical significance.

## Results

### NCT air-puff increased microbial colonies in aerosols close to the nozzle

**Fig 1:**
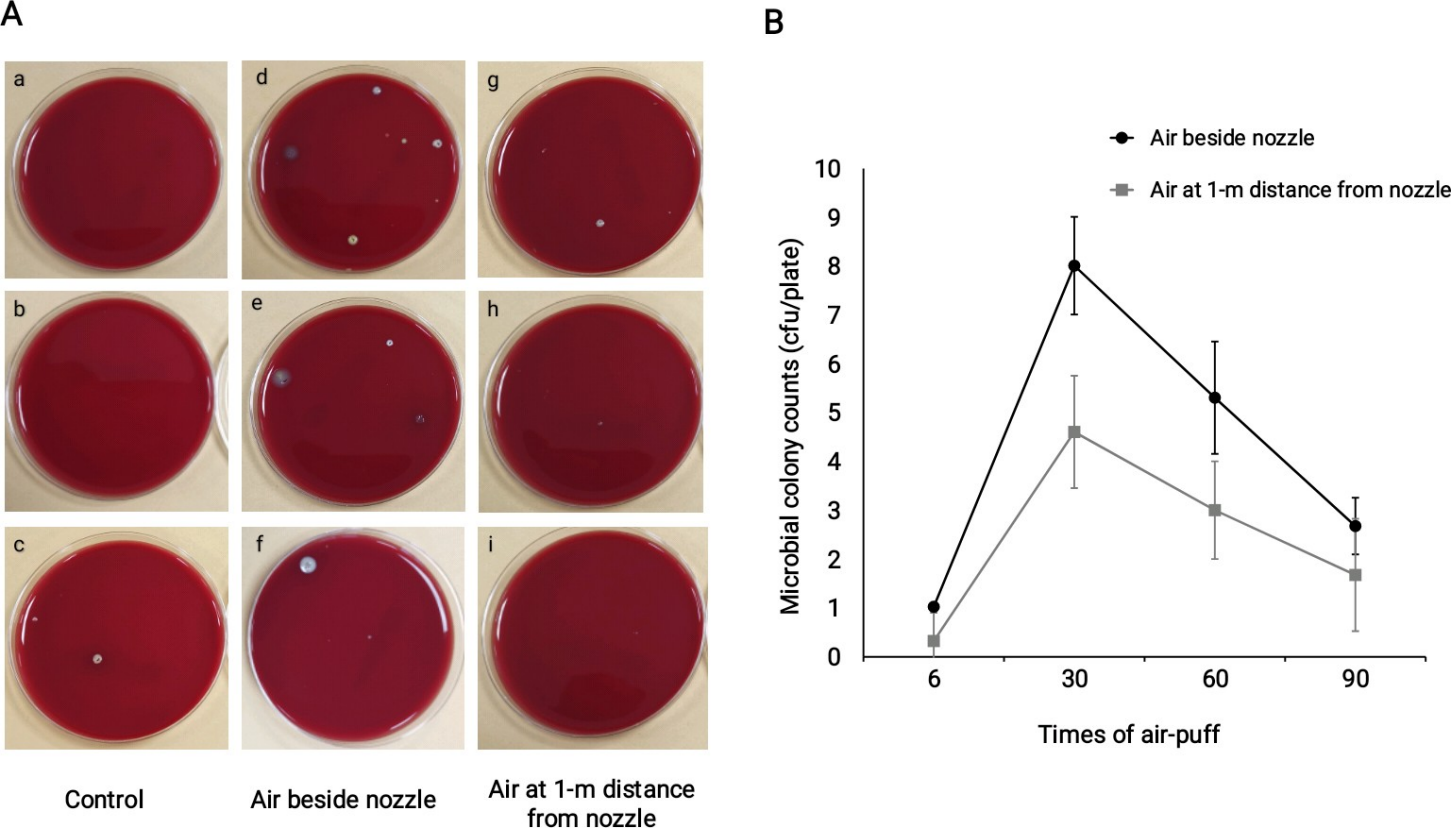
Increased microbial colonies were detected in air samples beside the nozzle after NCT air-puff. **A**: Representatives of culture plates at different sampling sites after NCT air-puff. a,b,c: room air samples after UV disinfection; d,e,f: air samples beside the nozzle after different times of air-puff; g,h,i: air samples at 1-m distance from the nozzle after different times of air-puff. **B**: More microbial colonies were detected in air samples beside the nozzle compared with samples at 1-m distance (overall difference *p*<0.05). For the difference between groups, a significant difference was observed in the group of 30 times air-puff (*p<*0.05). There were no significant differences between the other three groups.

After UV light disinfection, the average colony counts of the room air sample was 0.7 cfu/plate, which is below the reference level of 4.0 cfu/plate for a 15-m^2^ room according to the Hospital Disinfection and Sanitation standard of China (GB15982-2012). This indicates that the UV disinfection eliminated most of the microbes in the room air. Patients who visited the Department of Ophthalmology of the Qilu Hospital of Shandong University and have been excluded infectious eye diseases were examined with air-puffed NCT and then sampled. The average colony counts of the samples collected from air close to NCT nozzle and air at 1-m distance from the nozzle increased after NCT air-puffs (Fig 1A). The number of colony counts was higher in the air samples close to the NCT nozzle compared with that at a 1-m distance from the NCT nozzle after NCT air-puffs (means of 4.3 cfu/plate vs 2.4 cfu/plate, *p* = 0.001). The average colony number of air samples reached a peak in the group of 30 times air-puff and began to decline as the air-puff times increased to 60 and 90. For the difference between groups, a significant difference was observed in the group of 30 times air-puff (means of 8.0 cfu/plate vs 4.7 cfu/plate, *p* = 0.01). There were no significant differences between the other three groups (Fig 1B). The related original culture plate pictures and detailed colony counts were shown in S1 File and S1 Table. Species identification by mass spectrometry revealed 16 strains of microbes as shown in Table 1, all of which were non-pathogenic.

**Table 1:** Microbial strains identified by mass spectrometry in room air samples after UV light disinfection and air samples collected beside the nozzle and at 1-m distance from the nozzle after NCT air-puff.

Site	Times of air-puff	Microbial Strains
Room air after ultraviolet light disinfection	N/A	Micrococcus sp, Moraxella osloensis
Air samples beside the nozzle	6	Micrococcus luteus
	30	Micrococcus luteus, Escherichia coli, Kytococcus schroeteri, Pseudarthrobacter oxydans, Bacillus feed
	60	Pseudarthrobacter oxydans, Corynebacterium afermentans, Corynebacterium lipophiloflavum
	90	Staphylococcus xylosus Staphylococcus epidermidis
Air samples at 1-m distance from the nozzle	6	Staphylococcus epidermidis
	30	Micrococcus luteus, Bacillus littoral, Staphylococcus epidermidis
	60	Agrococcus jenensis, Shewanell baltica, Staphylococcus epidermidis
	90	Clostridium bharat

Interestingly, in contrast to the results from the air samples, no microbial colonies were detected for the swab samples from the surface of the NCT nozzle before and after NCT air-puff.

### Disinfection with 75% alcohol reduces microbial aerosols

**Fig 2:**
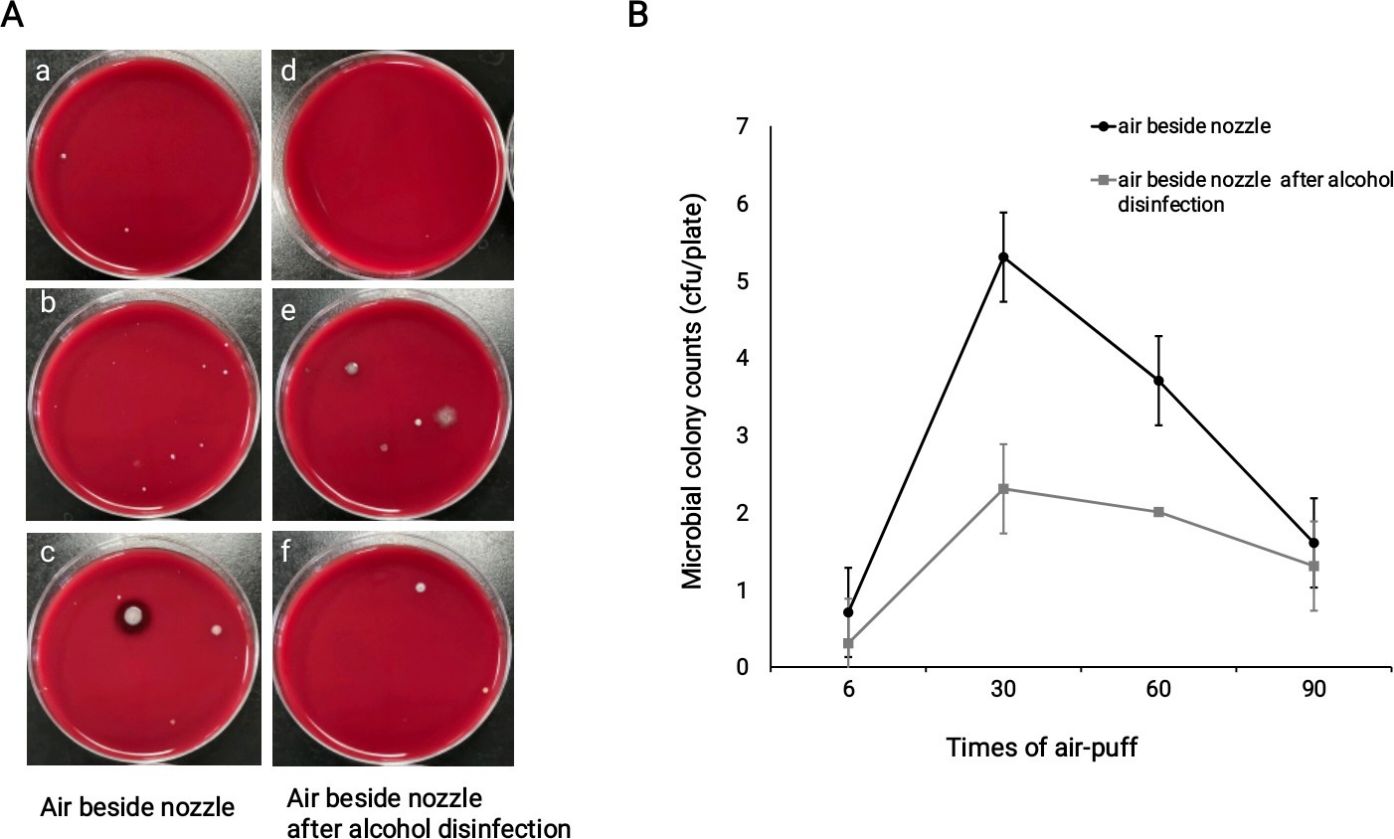
Less microbial colonies were detected in air samples beside the nozzle after 75% alcohol disinfection. **A**: Representatives of culture plates of air samples beside the nozzle after NCT air-puff before and after 75% alcohol disinfection. a,b,c: air samples besides nozzle after different times of air-puff; d.e.f: air samples besides nozzle after different times of air-puff with alcohol disinfection. **B**: Less microbial colonies were detected in air samples besides nozzle after alcohol disinfection (overall difference *p*<0.05). For the difference between groups, a significant difference was observed in both groups of 30- and 60- times air-puff (*p*<0.05). There were no significant differences between the other two groups.

To evaluate the effect of alcohol disinfection on the inhibition of microbes contained in the aerosols, air samples were collected before and after routine disinfection with 75% alcohol after NCT examination for the last patient in each group. As shown in Fig 2A, colonies were significantly reduced in the aerosols close to the NCT nozzle after disinfection with 75% alcohol compared with samples collected before 75% alcohol disinfection (overall means of 1.5 cfu/plate vs 2.8 cfu/plate, *p* = 0.003). For the difference between groups, a significant difference was observed in the group of 30- and 60- times air-puff (means of 2.3 cfu/plate vs 5.3 cfu/plate, *p* = 0.015; 2.0 cfu/plate vs 3.7 cfu/plate, *p* = 0.038) (Fig 2B). There were no significant differences between the other two groups. The related original culture plate pictures and detailed colony counts were shown in S1 File and S2 Table. Species identification by mass spectrometry revealed 12 strains of microbes as shown in Table 2, all of which were non-pathogenic.

**Table 2:** Microbial strains identified by mass spectrometry in air samples collected beside the nozzle after different times of air-puff with or without alcohol disinfection.

Site	Times of air-puff	Microbial Strains
Air beside nozzle	6	Staphylococcus xylosus
	30	Escherichia coli, Kytococcus schroeteri, Staphylococcus warneri, Bacillus cereus
	60	Paenarthrobacter ureafaciens, Bacillus cereus, Bacillus simplex, Staphylococcus hominis
	90	Staphylococcus epidermidis, Staphylococcus haemolyticus
air around jet after alcohol disinfection	6	none
	30	Staphylococcus warneri, Micrococcus luteas
	60	Staphylococcus hominis
	90	Staphylococcus haemolyticus

## Discussion

Previous studies have shown that NCT can produce aerosols, which could be one of the transmission routes of virus [11, 16]. In this study, we evaluated the microbial load in aerosols after air-puffed NCT examination and the effect of 75% alcohol disinfection on the inhibition of air-borne microbes. We detected an increase in microbial colony number in the air samples close to the nozzle after air-puffed NCT compared with samples at a 1-m distance from the NCT nozzle in the consultation room. This suggests that air-puffed NCT could be a potential source of the microbial aerosols that may lead to transmission of pathogens in the healthcare settings. Disinfection with 75% alcohol for the nozzle and air around it was efficient at decreasing the microbes contained in the aerosols and theoretically this prevention measure could also inhibit virus transmission. This will give guidance for the prevention of virus transmission and protection of hospital staff and patients during the great pandemics of COVID-19 worldwide.

Only 0.7 cfu/plate was detected from the room air samples and no microbial colony was detected from the surface samples of the NCT nozzle after 15 minutes of disinfection with UV. This indicates that UV disinfection eradicated most microorganisms in the consultation room. UV disinfection has served as the most effective sterilization method in hospitals with sterilization efficacy of 99.9%. Studies on both SARS and MERS have shown that UV light could inactivate these viruses [19, 20]. Therefore, we assume that UV light may have a similar effect on SARS-CoV-2. During the COVID-19 pandemic in China, UV light has been widely used in hospitals to disinfect rooms and personal protective equipment. However, UV irradiation may damage human DNA, causing health problems such as skin cancer, cornea damage, or cataracts in the eye.

We noted that the presence of patients in the ophthalmic consultation room was associated with an increase in microbial load in the room air. We found that the number of colony count was high in the air samples close to the NCT nozzle compared with that at a 1-m distance from the NCT nozzle after 6 times of air-puff examination per patient. A significant difference was observed after 30 times of air-puff examination of 5 patients. However, the average colony count of air samples decreased as the air-puffed NCT times increased to 60 and 90. This may be due to the increased air ventilation with more patients’ activities in the room. Thus, we speculated that the microbial aerosols were produced after the air-puff but they were localized in the air close to the NCT nozzle and tend to spread and attenuate with air ventilation. This is consistent with a study by Li et al. which showed that aerosol particle formation fluctuated with the increase of times of air-puff and that aerosol accumulation was higher in the hall with insufficient air ventilation [17]. Species identification showed that all the culture strains isolated were non-pathogenic and common in air, human skin, or ocular surface. All the patients who underwent NCT examination used facial masks during the whole course to avoid confounding microbes originating from the respiratory tract. Our results suggest that NCT may be a potential source of aerosols containing microbes that could lead to transmission of pathogens. Hence, an NCT examination should be avoided, if not necessary, during the pandemic of COVID-19. Tonometer should be used in ventilated areas and the time interval between patients should be extended. Using facemask is recommended during the NCT examination.

In contrary to the results of the air samples, no microbial colonies were detected for the swab samples from the surface of the NCT nozzle before and after the air-puffed NCT. No microbial cultures could be detected even when two cotton swabs with different cotton head sizes were used to scrub the surfaces around the NCT nozzle in order to increase sampling size and amount. This demonstrated that the microbial aerosols formed during the air-puff by NCT were likely suspended and disseminated in the air. However, aerosol particles may be precipitated on the surfaces around the NCT nozzle after a prolonged time.

Disinfection with alcohol is effective against bacteria, fungi and most coated-viruses because alcohol denatures the protective protein coat. Therefore, a proper concentration of alcohol is widely preferred for disinfection in hospitals and during viral outbreaks. In the present study, we sprayed 75% alcohol on the NCT nozzle and the surrounding air and wiped the nozzle surface using 75% alcohol. Our results showed that colonies were significantly reduced in the aerosols close to the NCT nozzle after disinfection with 75% alcohol compared with samples before disinfection, indicating that 75% alcohol disinfection is efficient in inhibiting the microbial aerosols formed during the air-puffed NCT. It has been shown that SARS-CoV-2 can effectively be deactivated by alcohol, ethanol and 2-propanol [21, 22]. Our results add to the existing evidence that alcohol disinfection may be theoretically effective in preventing the spread of viruses and thus could be essential in minimizing infection during NCT examinations. The American Academy of Ophthalmology recommended that 70% of alcohol solutions are effective in disinfecting tonometer tips from SARS-CoV-2 [23, 24]. These findings also apply to slit lamps and other diagnostic equipment, which are susceptible to patient-specialist contact. Disinfecting the NCT with alcohol after every examination is highly recommended. However, disinfection with alcohol sprays should be applied cautiously because alcohol contains highly volatile and flammable compounds. In addition, 70% alcohol or other disinfectants may damage the tonometer prisms, therefore, the prisms should be regularly examined.

This study has several limitations. First, it was a pilot study with limited sample size in a single center. Second, since the door of the examination room remained open to increase ventilation during high patients’ traffic, this could have compromised our outcomes. Third, the enrolled patients were chosen randomly and thus different population of the patients might have influenced the microbial species. For future research, it’s necessary to increase the sample size and involve multiple ophthalmic centers to verify the findings of the current study. At the same time, we suggest more in-depth studies on the identification and quantification of contagious bio-aerosols in department of ophthalmology as well as other high-risk medical environment such as department of dentistry, operating rooms and endoscopy units. It’s also important to further analyze the risks for healthcare stuffs including ophthalmologists and dentists with continuous exposure to bio-aerosols generated in specific medical procedures for better awareness and prevention strategies.

In summary, microbial aerosols were produced around the nozzle by NCT air-puff in the ophthalmic consultation room, which could be one of the transmission routes of viruses in the departments of ophthalmology. Disinfection with 75% alcohol was efficient in suppressing microbial aerosols and theoretically this prevention measure could also inhibit virus transmission. These findings support the need for strict adherence to hygiene and routine disinfection in hospitals. This is fundamental in the control and prevention of viral transmission and protection of both the healthcare service providers and the patients during and after the prevalence of COVID-19.

## Supporting information

S1 File The original culture plate picture data. (ZIP)

S1 Table Detailed colony counts (cfu/plate) in culture plates of air samples at different sampling sites after predetermined times of NCT air-puff in three repeated experiments. (DOCX)

S2 Table Detailed colony counts (cfu/plate) in culture plates of air samples beside the nozzle after predetermined NCT air-puff before and after 75% alcohol disinfection in three repeated experiments. (DOCX)
