# Supplementary figures and images for: Increased microbial loading in aerosols produced by non-contact air-puff tonometer and relative suggestions for the prevention of coronavirus disease 2019 (COVID-19)
Source: PLoS One. 2020 Oct 8;15(10):e0240421. doi: 10.1371/journal.pone.0240421 (PMC7544126; doi:10.1371/journal.pone.0240421)

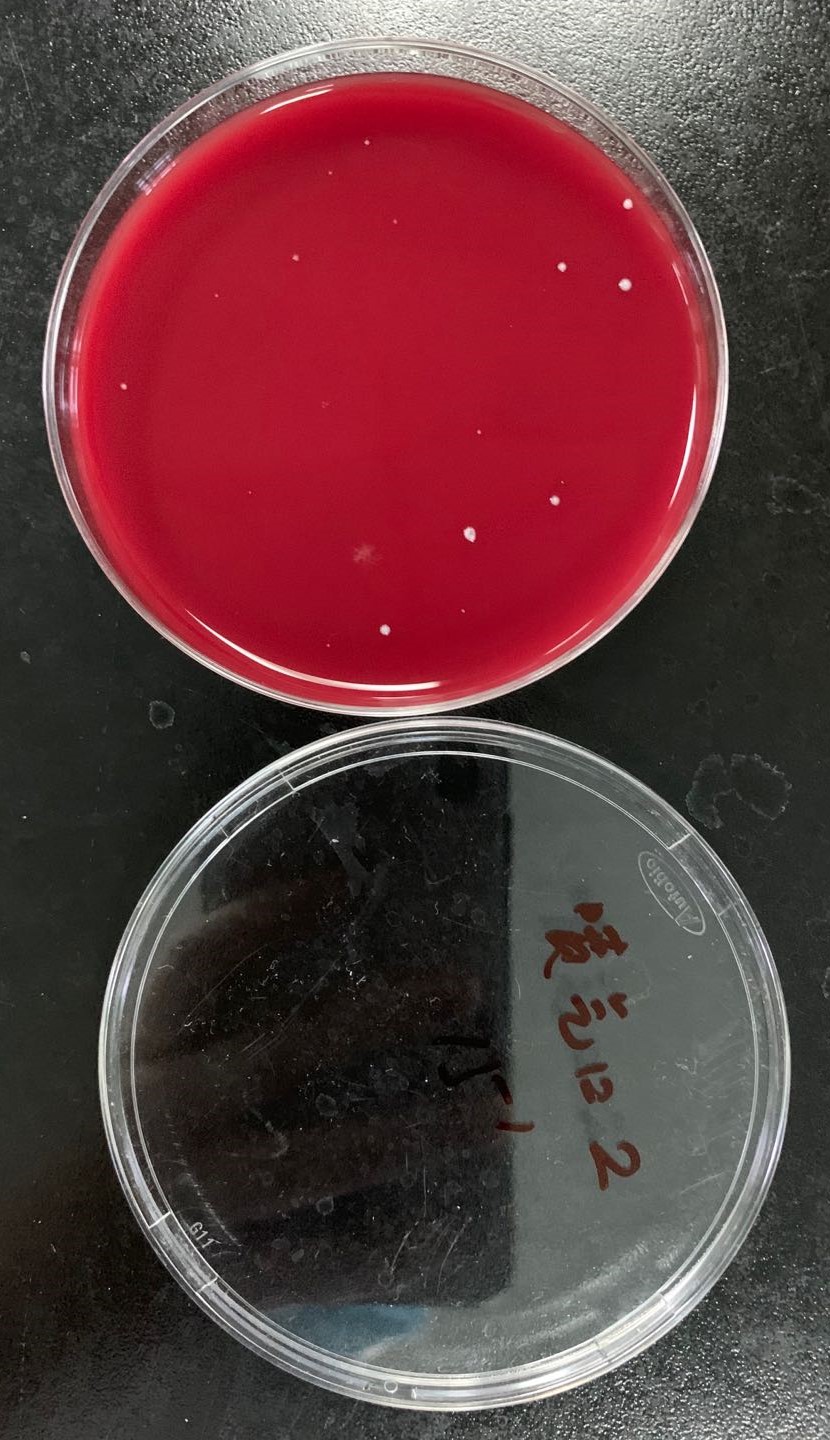

Supplement: S1 File — (ZIP) [file pone.0240421.s001.zip › S1 File/Figure2--air beside nozzle before disinfection 2.jpg]

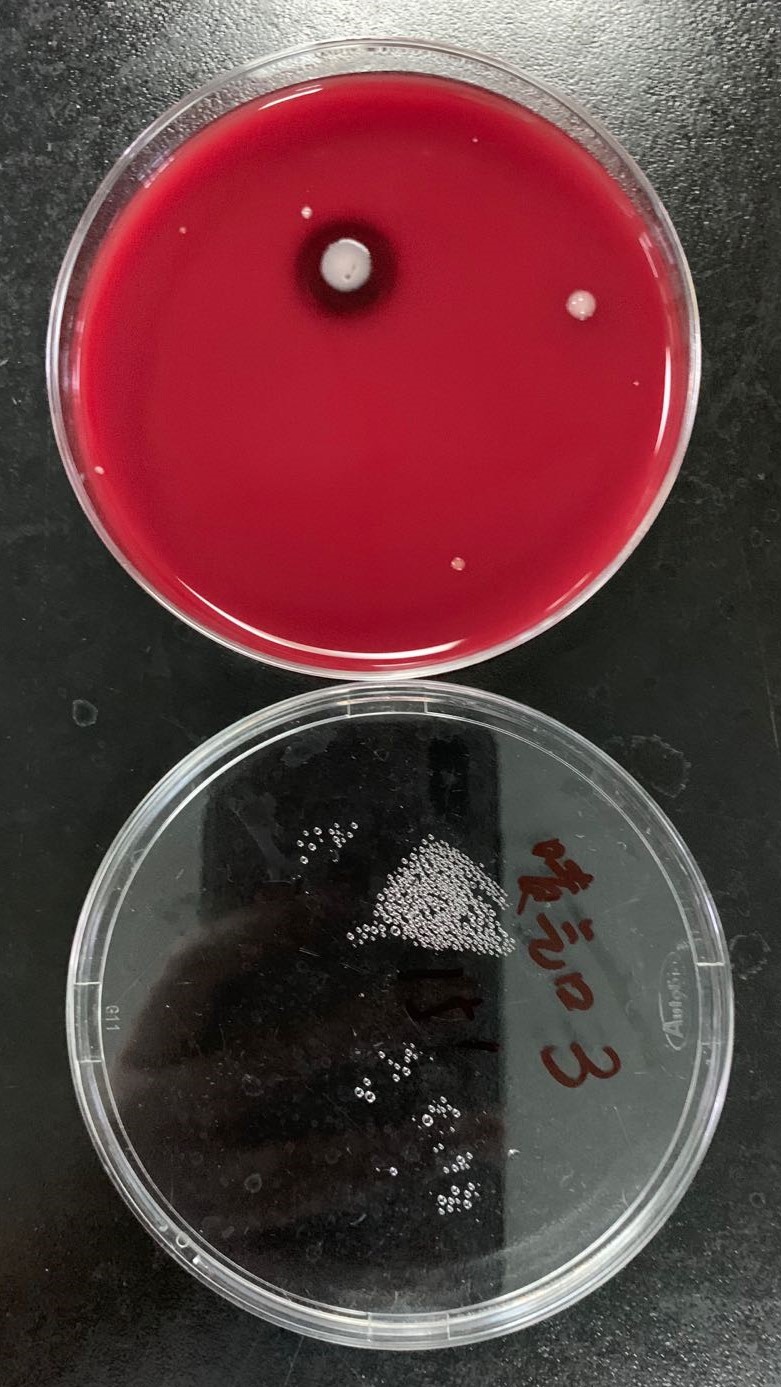

Supplement: S1 File — (ZIP) [file pone.0240421.s001.zip › S1 File/Figure2--air beside nozzle before disinfection 3.jpg]

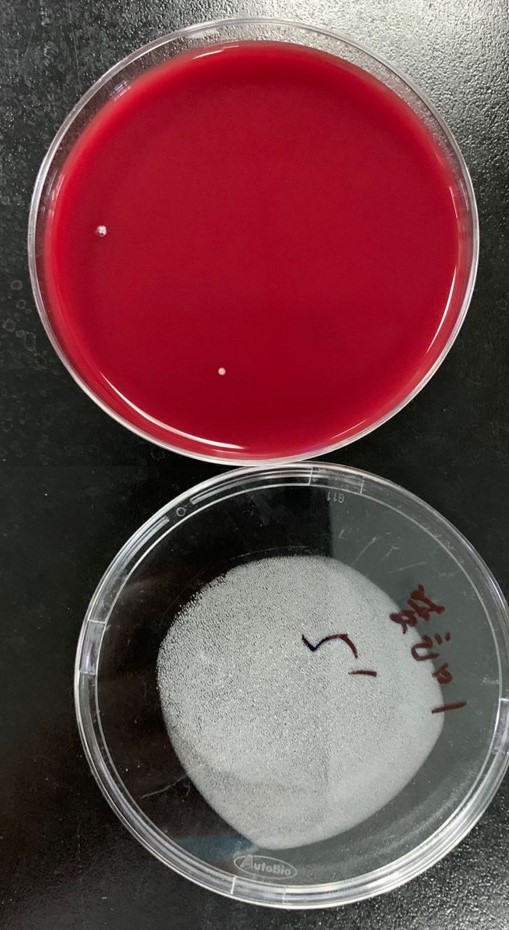

Supplement: S1 File — (ZIP) [file pone.0240421.s001.zip › S1 File/Figure2--air beside nozzle before disinfection 1.jpg]

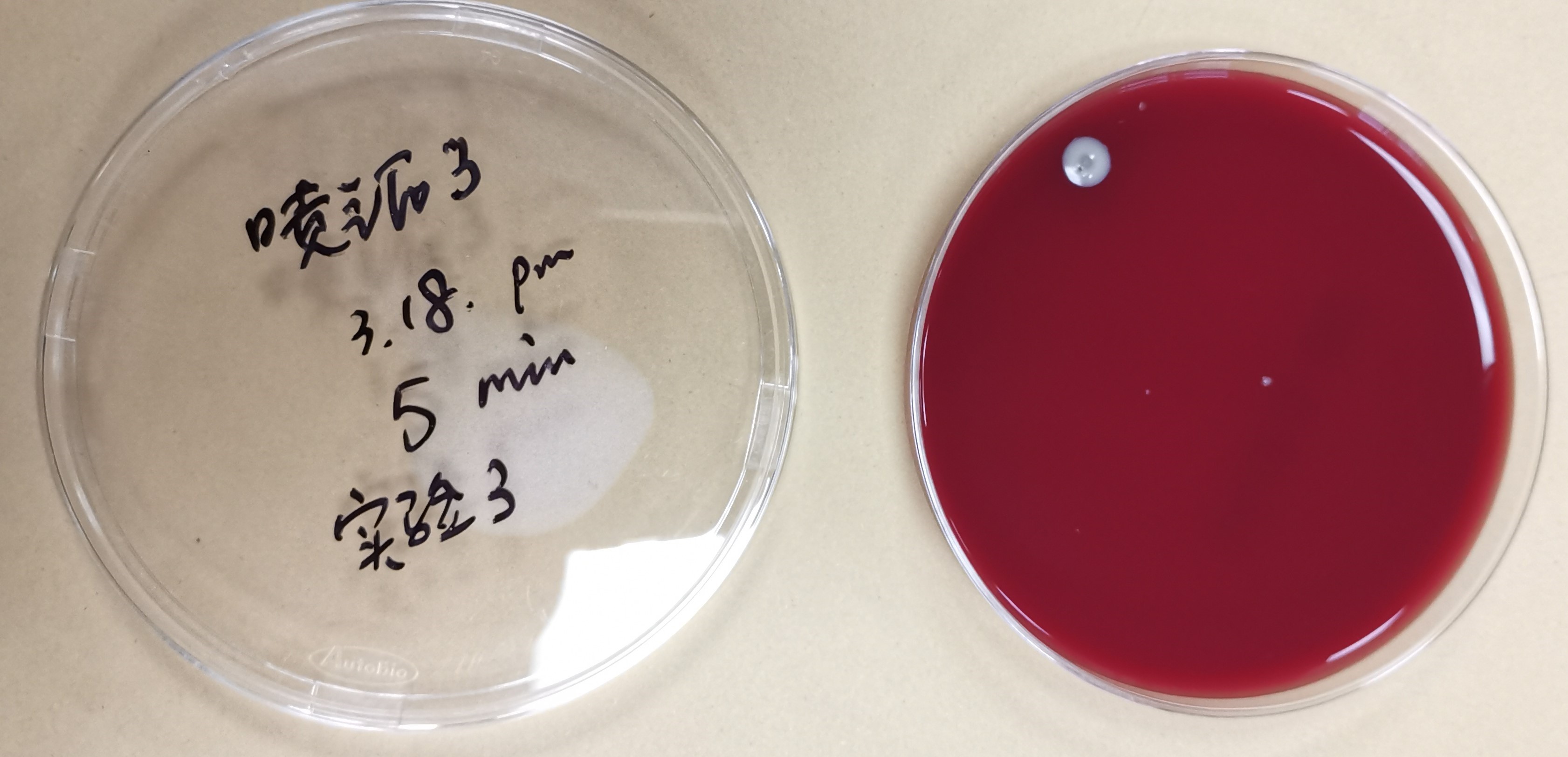

Supplement: S1 File — (ZIP) [file pone.0240421.s001.zip › S1 File/Figure1-air beside nozzle 3.jpg]

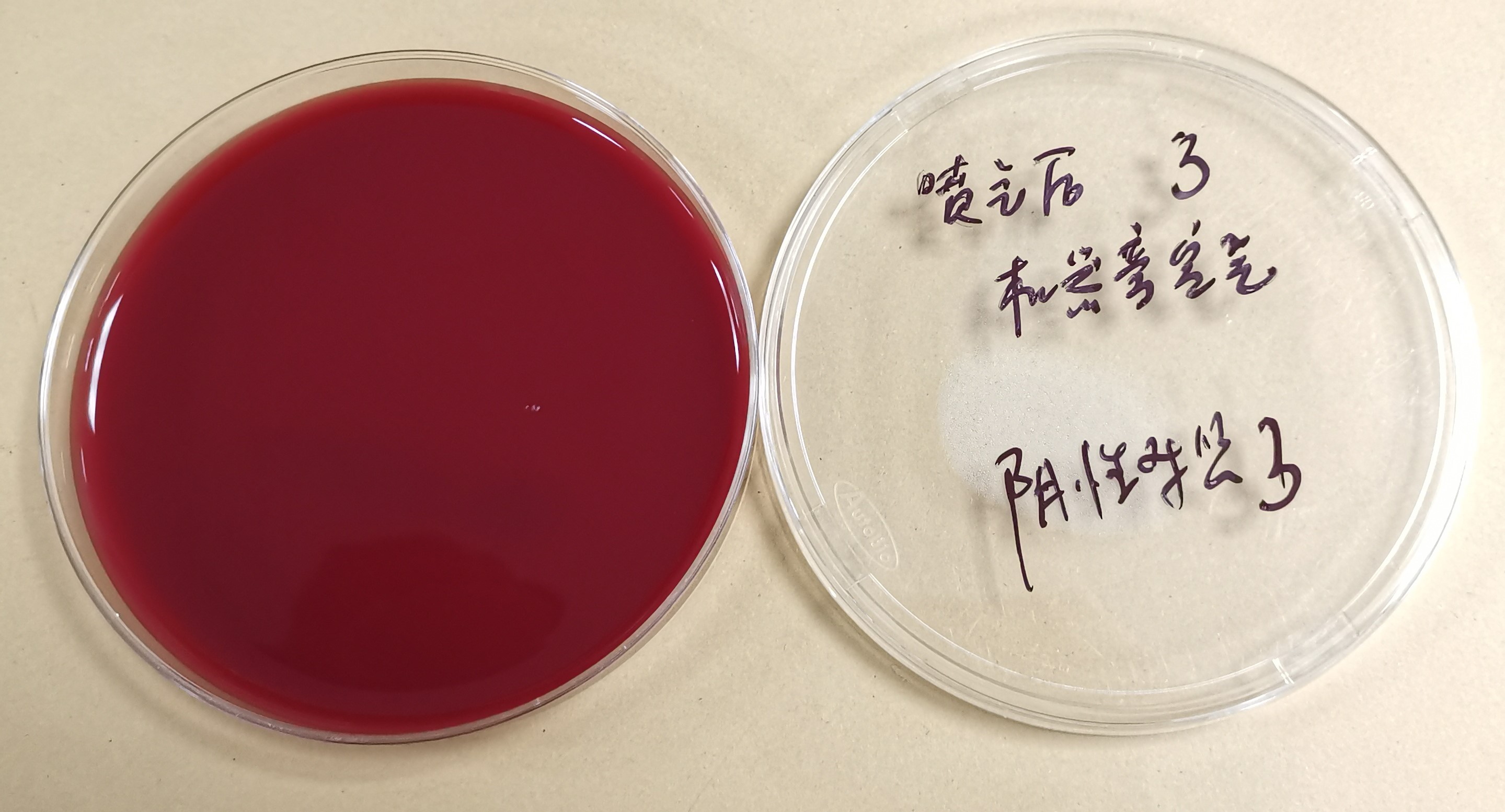

Supplement: S1 File — (ZIP) [file pone.0240421.s001.zip › S1 File/Figure1-air at 1-m distance 3.jpg]

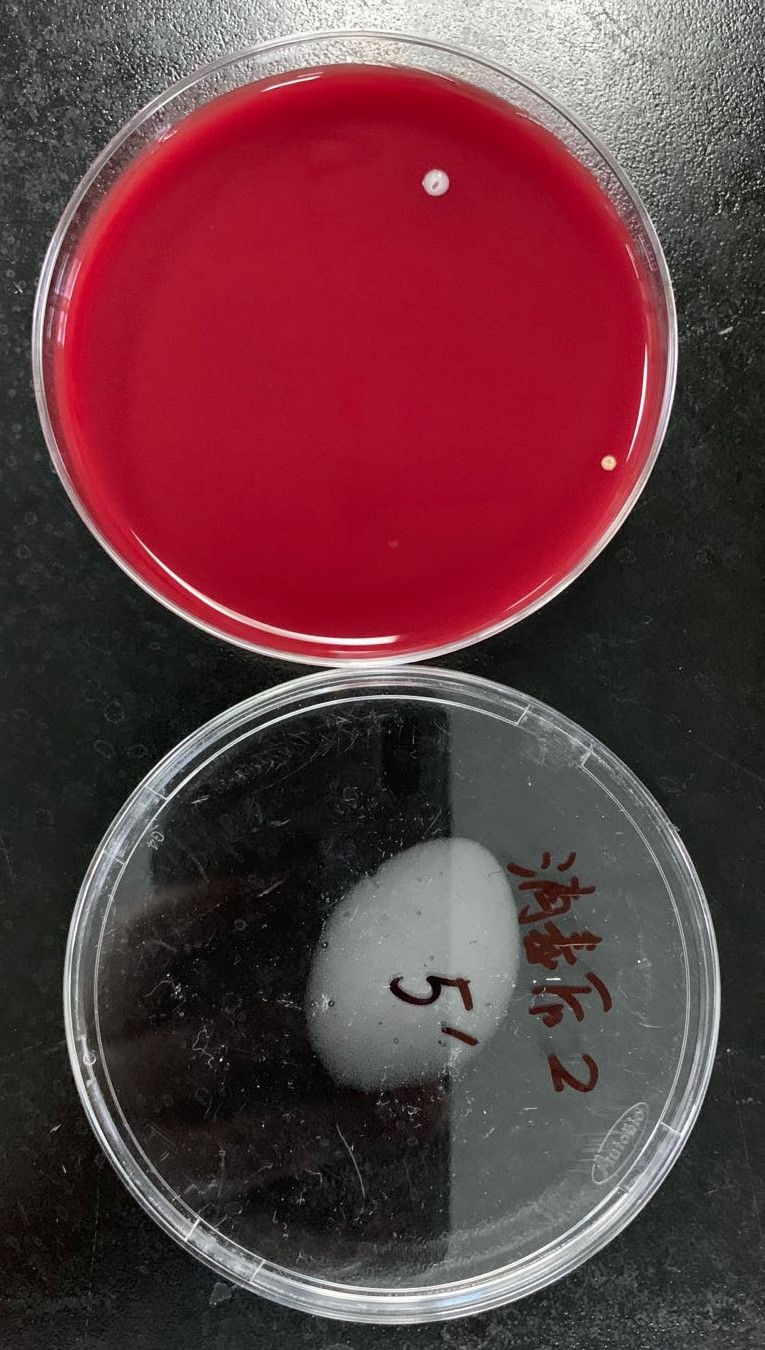

Supplement: S1 File — (ZIP) [file pone.0240421.s001.zip › S1 File/Figure2--air beside nozzle after disinfection 3.jpg]

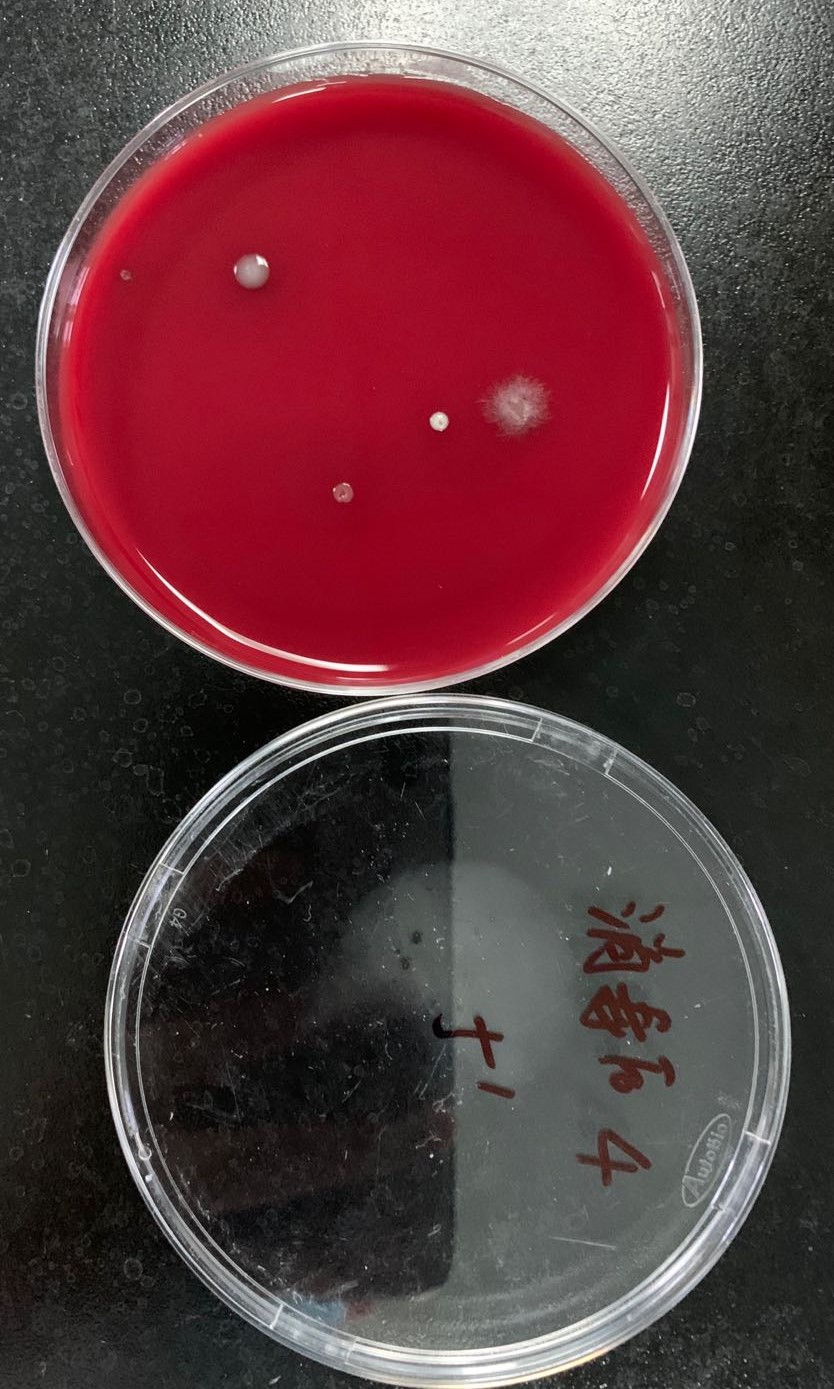

Supplement: S1 File — (ZIP) [file pone.0240421.s001.zip › S1 File/Figure2--air beside nozzle after disinfection 2.jpg]

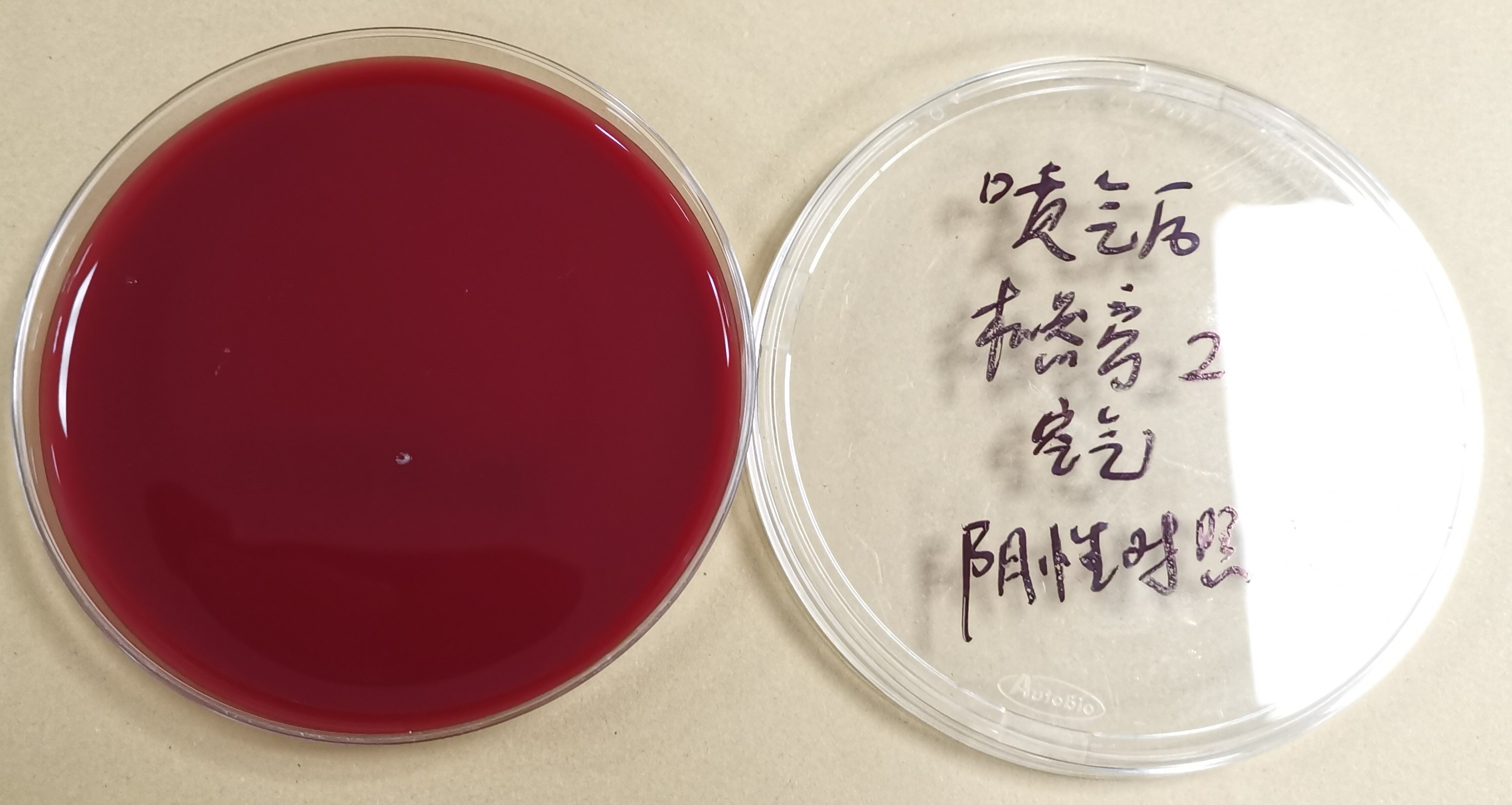

Supplement: S1 File — (ZIP) [file pone.0240421.s001.zip › S1 File/Figure1-air at 1-m distance 2.jpg]

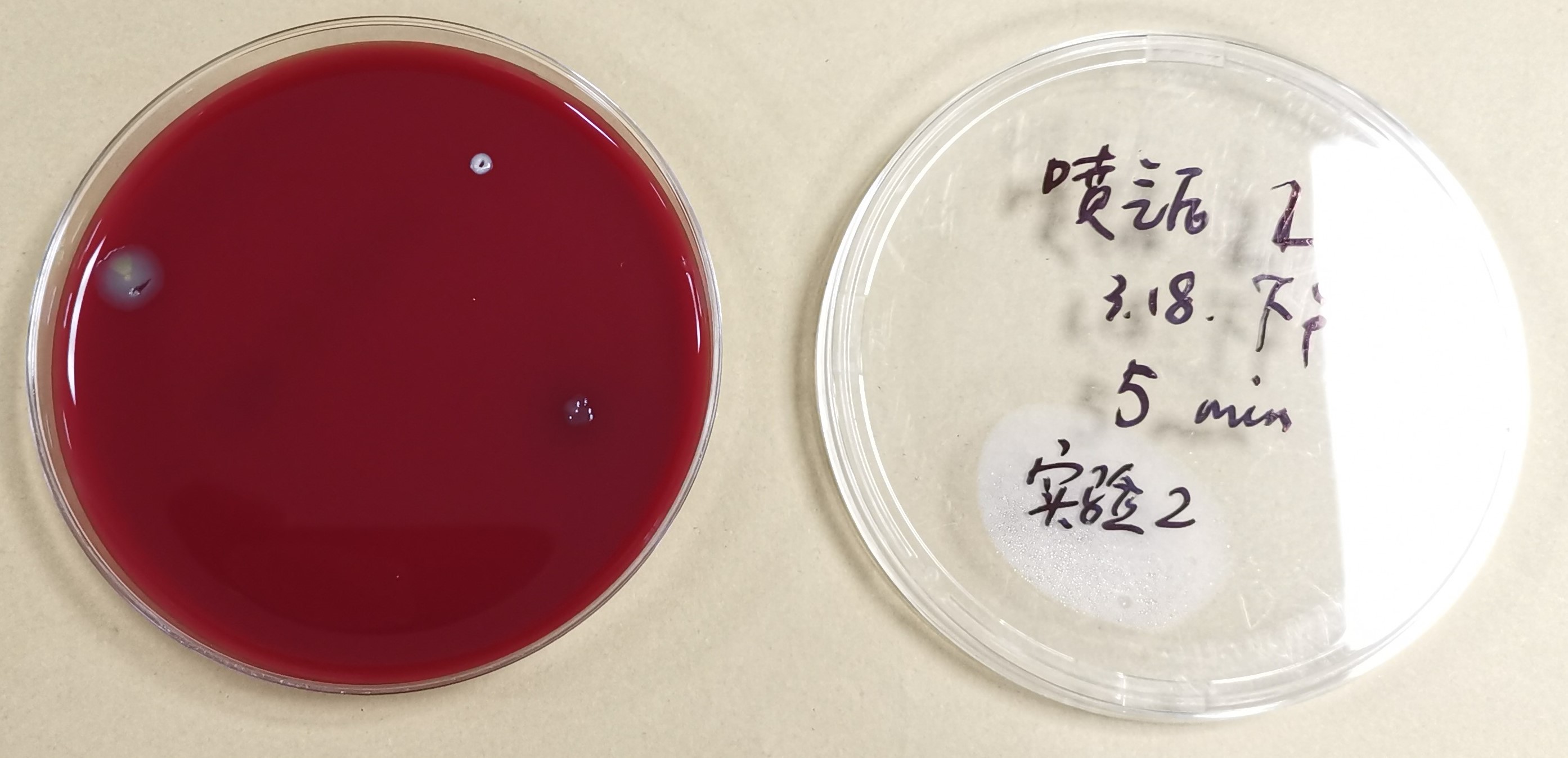

Supplement: S1 File — (ZIP) [file pone.0240421.s001.zip › S1 File/Figure1-air beside nozzle 2.jpg]

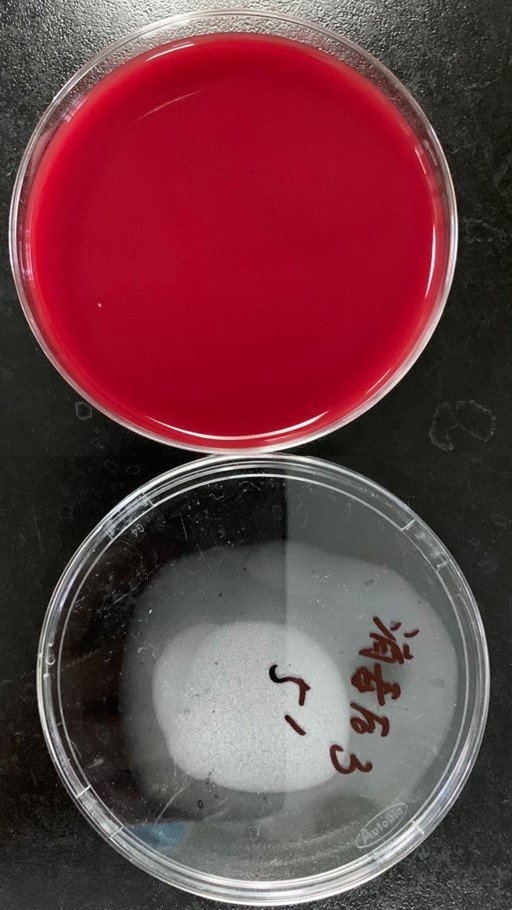

Supplement: S1 File — (ZIP) [file pone.0240421.s001.zip › S1 File/Figure2--air beside nozzle after disinfection 1.jpg]

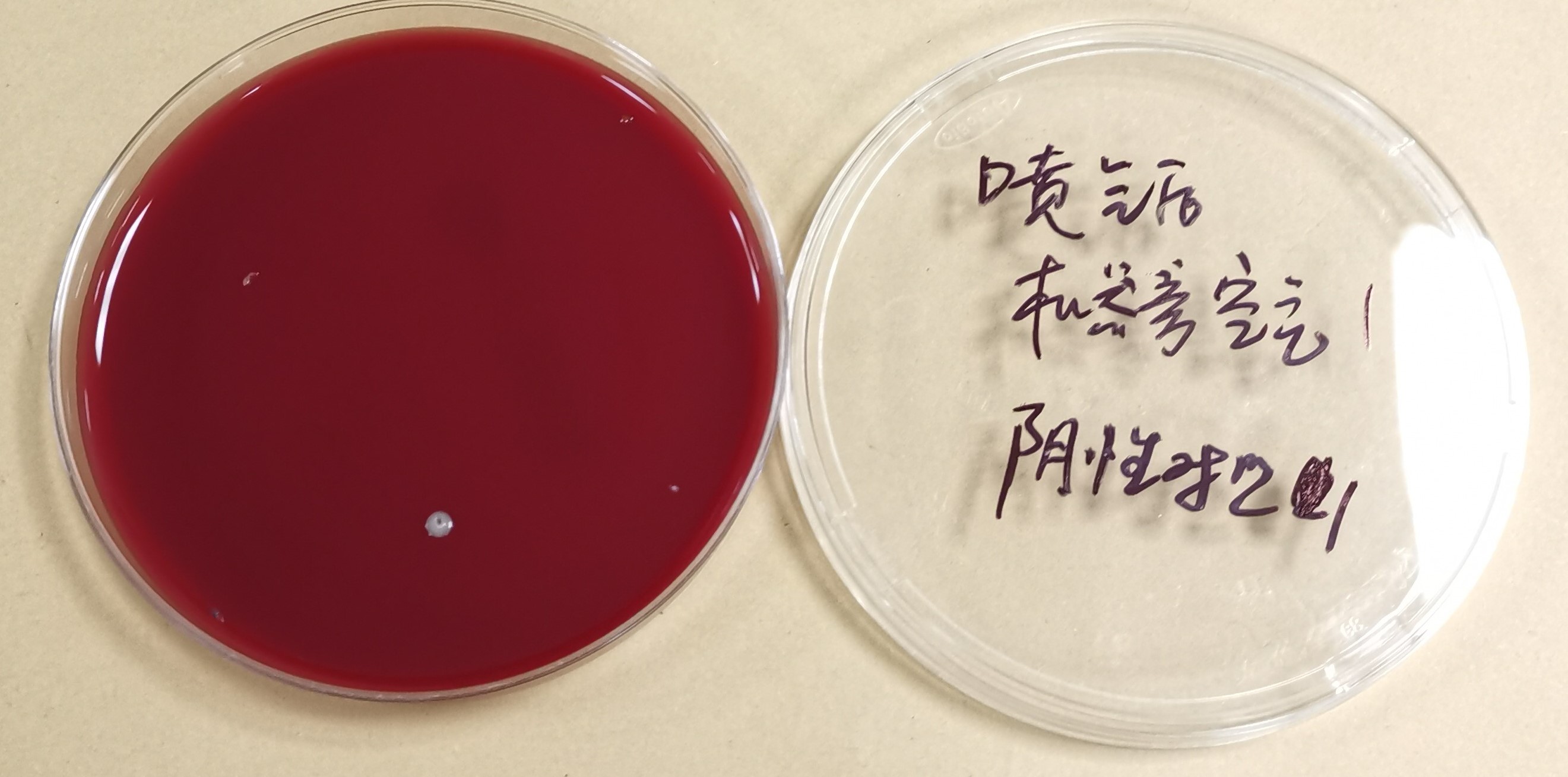

Supplement: S1 File — (ZIP) [file pone.0240421.s001.zip › S1 File/Figure1-air at 1-m distance 1.jpg]

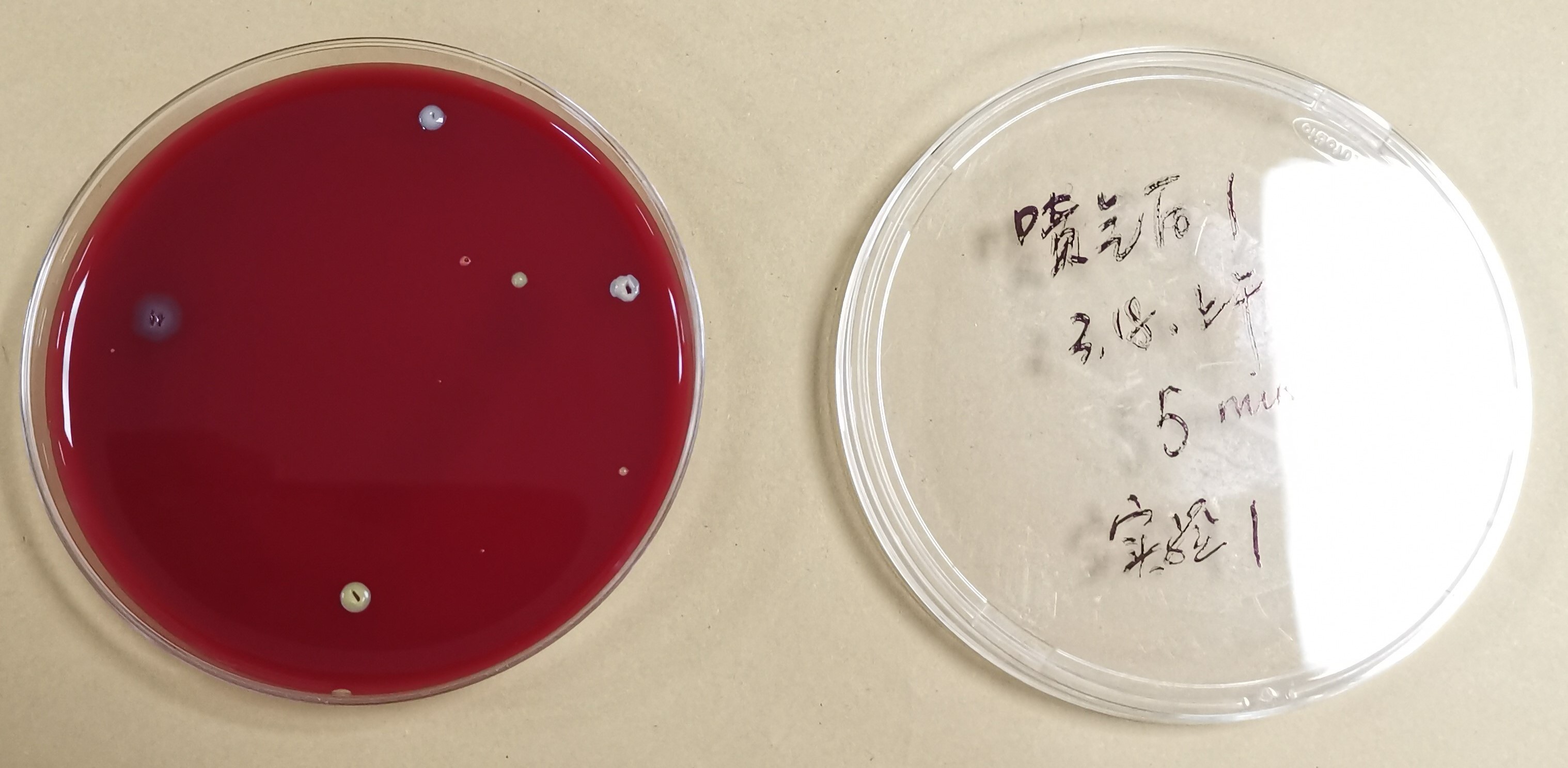

Supplement: S1 File — (ZIP) [file pone.0240421.s001.zip › S1 File/Figure1-air beside nozzle 1.jpg]

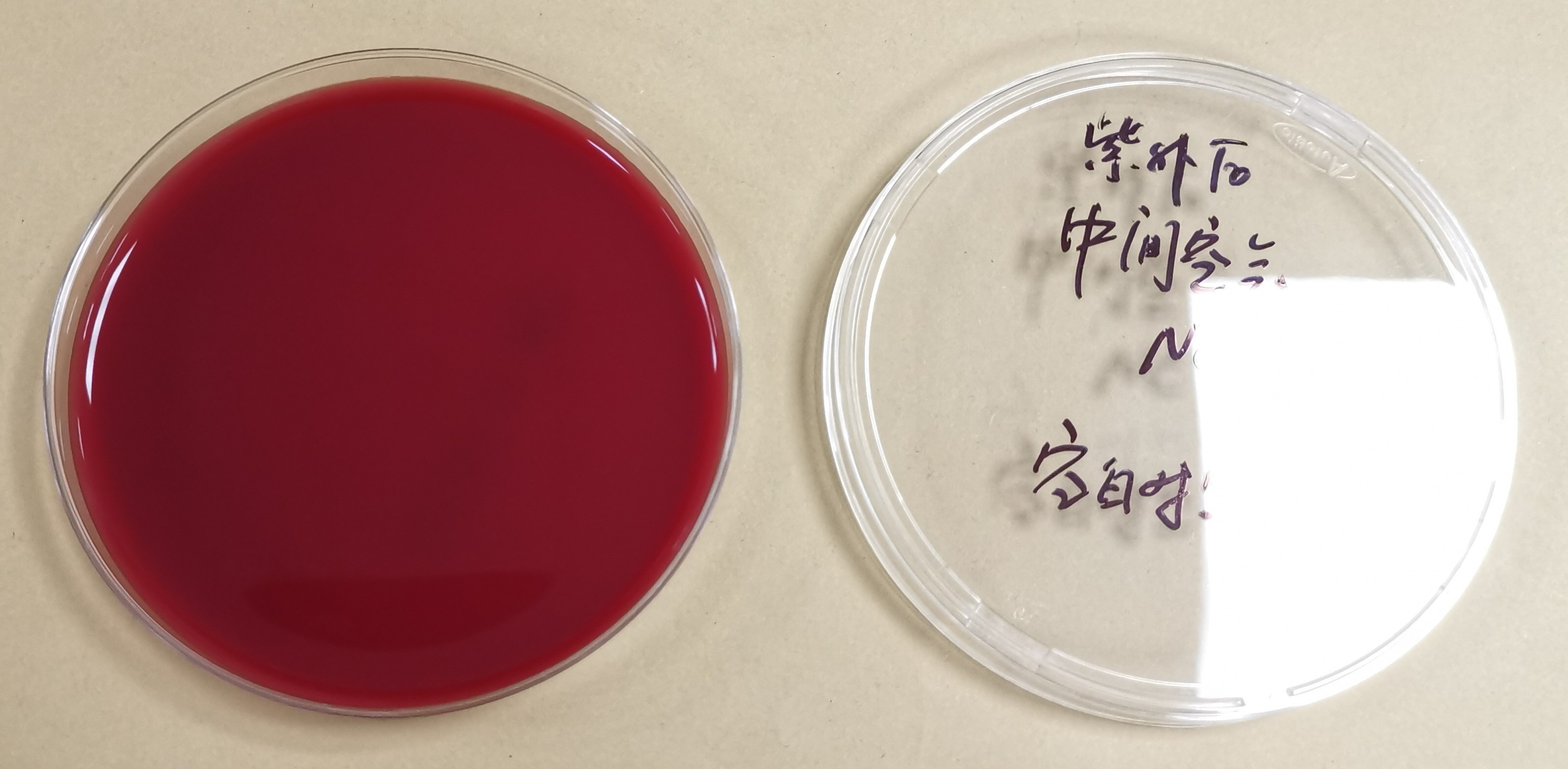

Supplement: S1 File — (ZIP) [file pone.0240421.s001.zip › S1 File/Figure1-control1.jpg]

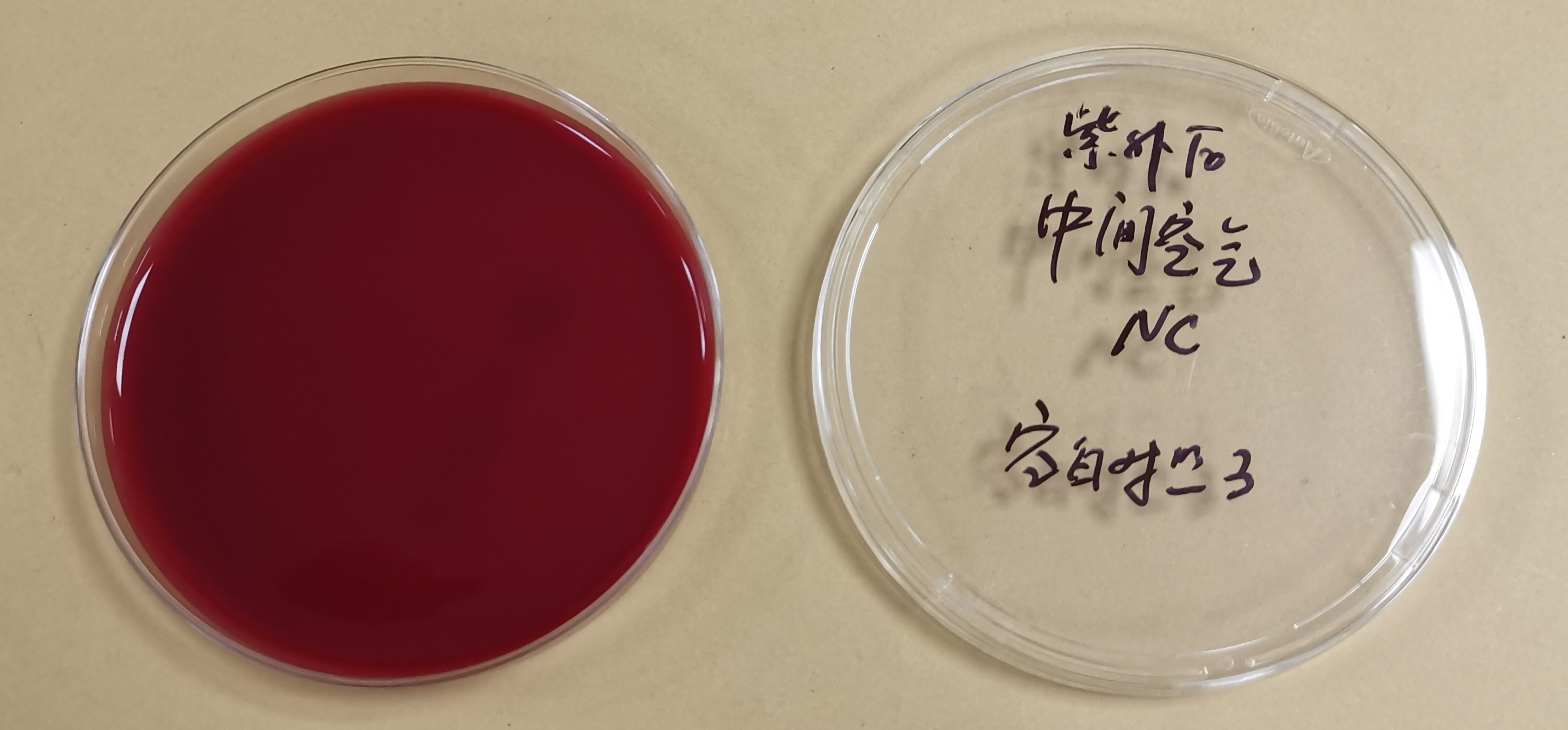

Supplement: S1 File — (ZIP) [file pone.0240421.s001.zip › S1 File/Figure1-control3.jpg]

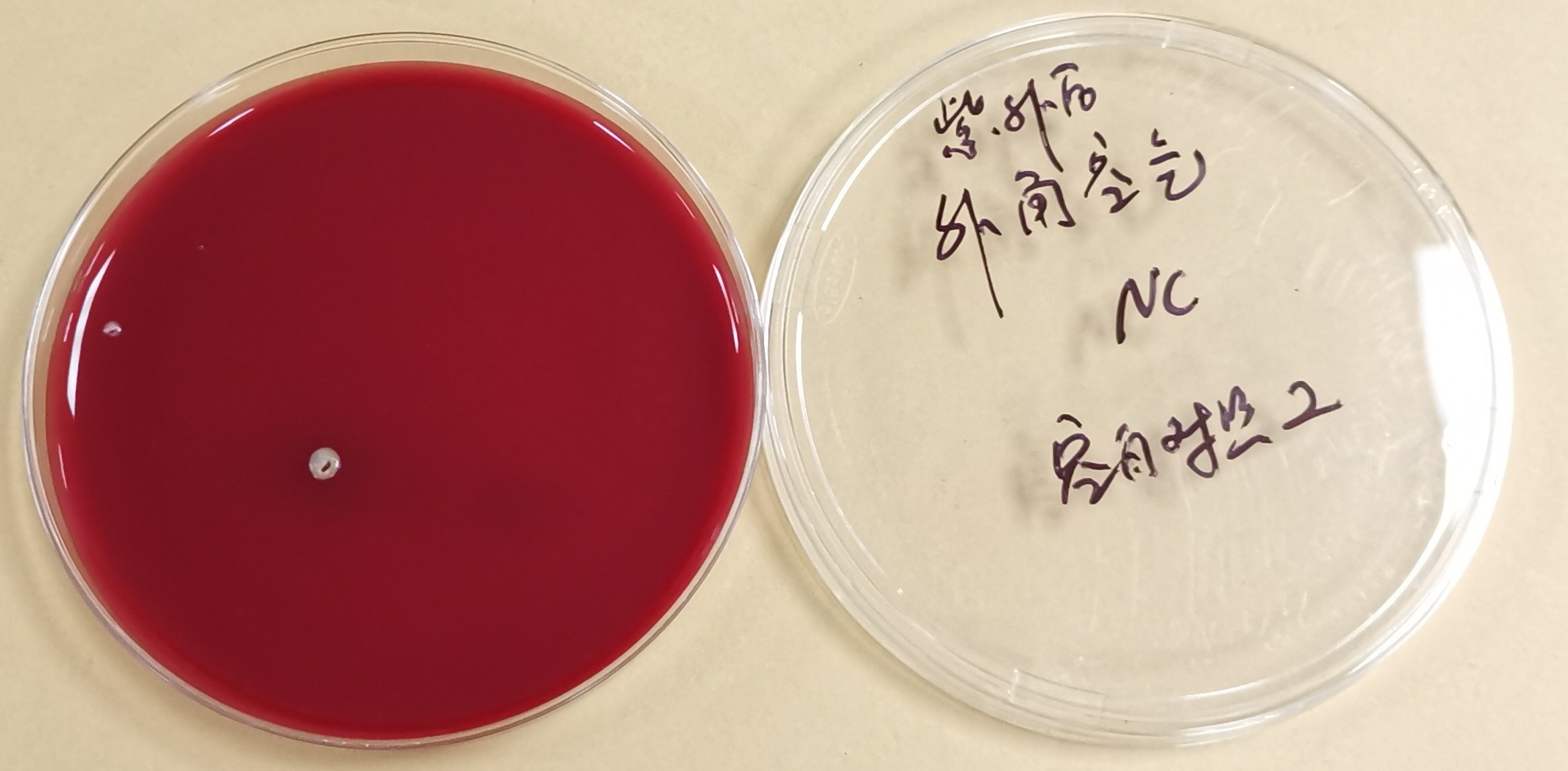

Supplement: S1 File — (ZIP) [file pone.0240421.s001.zip › S1 File/Figure1-control2.jpg]
