# Supplementary material for: Increased microbial loading in aerosols produced by non-contact air-puff tonometer and relative suggestions for the prevention of coronavirus disease 2019 (COVID-19)
Source: PLoS One. 2020 Oct 8;15(10):e0240421. doi: 10.1371/journal.pone.0240421 (PMC7544126; doi:10.1371/journal.pone.0240421)
Supplement: S1 Table — (DOCX) [file pone.0240421.s002.docx]

S1 Table. Detailed colony counts (cfu/plate) in culture plates of air samples at different sampling sites after predetermined times of NCT air-puff in three repeated experiments.

| **Times of puff** | **6 times** | **30 times** | **60 times** | **90 time** |
| --- | --- | --- | --- | --- |
| **Air besides nozzle** | 1 | 7 | 4 | 2 |
|  | 1 | 9 | 6 | 3 |
|  | 1 | 8 | 6 | 3 |
| **Average (Air besides nozzle)** | 1.00 | 8.00 | 5.33 | 2.67 |
| **Air at 1-m distance from nozzle** | 0 | 4 | 3 | 1 |
|  | 1 | 6 | 4 | 3 |
|  | 0 | 4 | 2 | 1 |
| **Average (Air at 1-m distance from nozzle)** | 0.33 | 4.67 | 3.00 | 1.67 |
