# Supplementary material for: Increased microbial loading in aerosols produced by non-contact air-puff tonometer and relative suggestions for the prevention of coronavirus disease 2019 (COVID-19)
Source: PLoS One. 2020 Oct 8;15(10):e0240421. doi: 10.1371/journal.pone.0240421 (PMC7544126; doi:10.1371/journal.pone.0240421)
Supplement: S2 Table — (DOCX) [file pone.0240421.s003.docx]

S2 Table. Detailed colony counts (cfu/plate) in culture plates of air samples beside the nozzle after predetermined NCT air-puff before and after 75% alcohol disinfection in three repeated experiments.

| **Times of puff** | **6 times** | **30 times** | **60 times** | **90 times** |
| --- | --- | --- | --- | --- |
| **Air besides nozzle** | 1 | 5 | 4 | 2 |
|  | 1 | 6 | 3 | 2 |
|  | 0 | 5 | 4 | 1 |
| **Average (Air besides nozzle)** | 0.67 | 5.33 | 3.67 | 1.67 |
| **Air besides nozzle after alcohol disinfection** | 0 | 2 | 2 | 1 |
|  | 1 | 3 | 2 | 2 |
|  | 0 | 2 | 2 | 1 |
| **Average (Air besides nozzle after alcohol disinfection)** | 0.33 | 2.33 | 2.00 | 1.33 |
